# Supplementary material for: Melatonin Alleviates Lipopolysaccharide-Induced Abnormal Pregnancy through MTNR1B Regulation of m6A
Source: Int J Mol Sci. 2024 Jan 5;25(2):733. doi: 10.3390/ijms25020733 (PMC10815701; doi:10.3390/ijms25020733)
Supplement: Supplementary file 1 [file ijms-25-00733-s001.zip › ijms-2759945-supplementary.pdf]

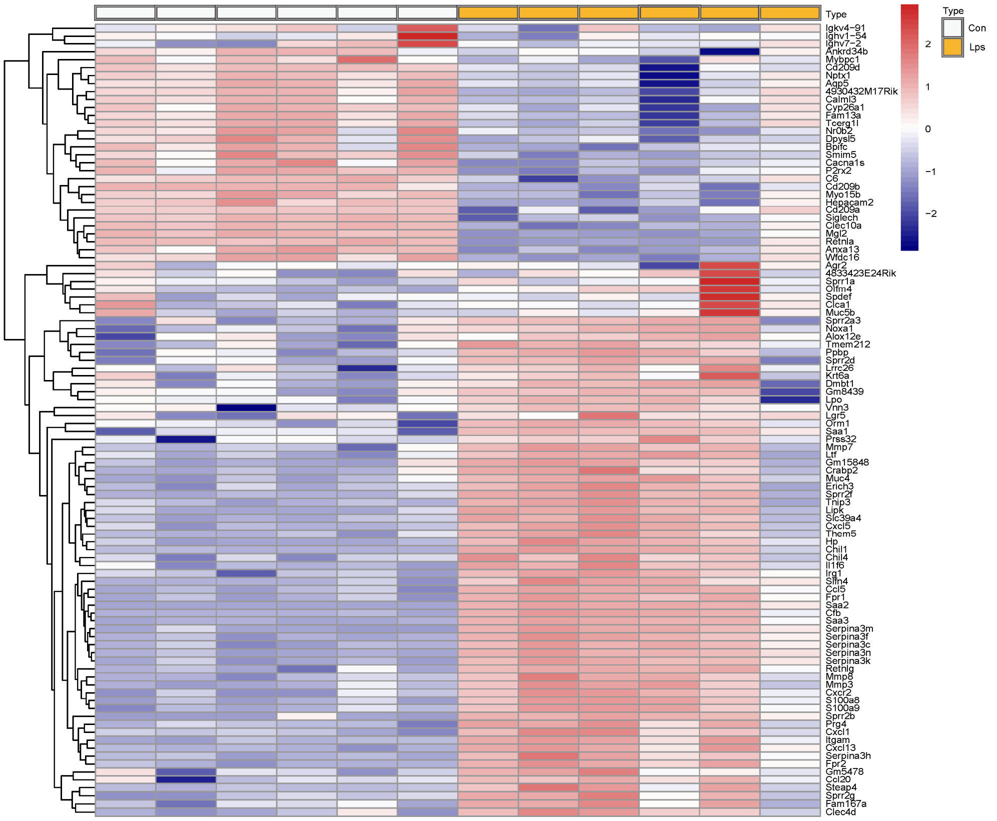

Figure S1 The differentially expression genes in decidua of mice in LPS challenge. Heatmap of differentially expressed genes (DEGs) in GEO datasets. The heatmap shows the top 100 genes with the most significant DEGs between the endometrium of the Con and LPS (Con: control group, Lps: LPS challenge group). n=3 independent biological replicates.

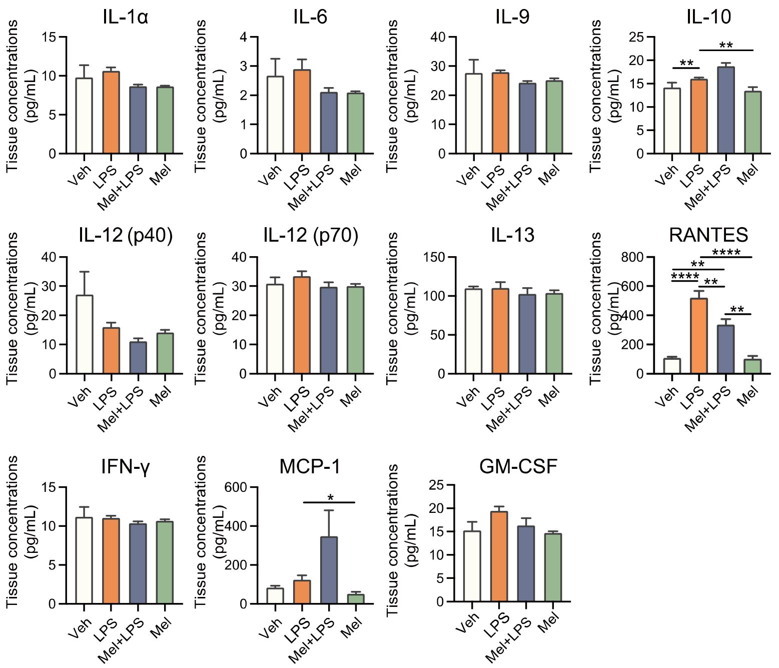

Figure S2 Mice uterus cytokine analysis by Luminex. n=4 independent biological replicates. Veh: Vehicle treatment group; LPS: LPS treatment group; Mel+LPS: Melatonin and LPS co-treatment group; Mel: Melatonin treatment group; The data are presented as the mean ± SD. Levels of statistical significance for all data were determined by one-way ANOVA and Tukey test (* Indicates significant difference between the two groups; *$p < 0.05$; **$p < 0.01$; ***$p < 0.001$; ****$p < 0.0001$).

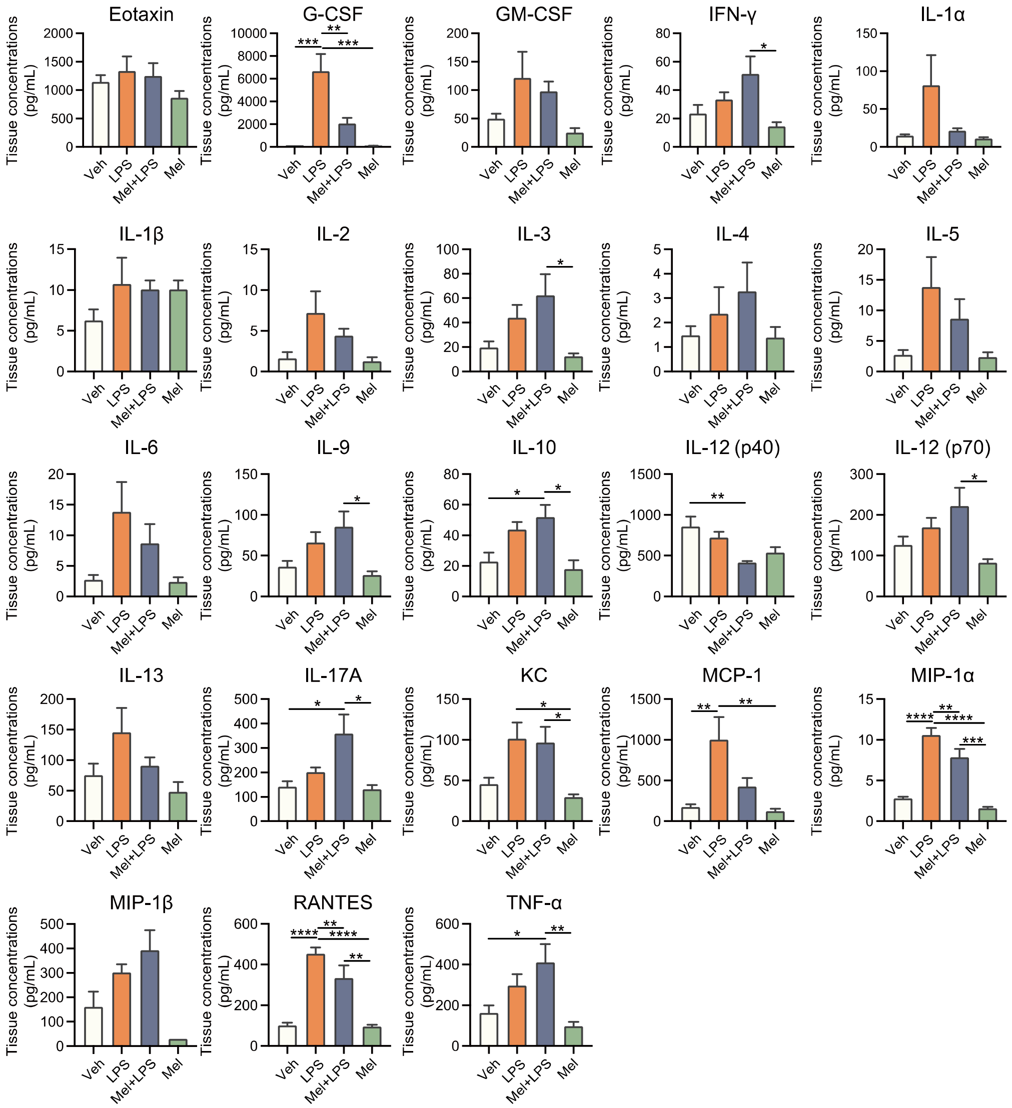

Figure S3 Serum cytokine analysis by Luminex. n=4 independent biological replicates. Veh: Vehicle treatment group; LPS: LPS treatment group; Mel+LPS: Melatonin and LPS co-treatment group; Mel: Melatonin treatment group; The data are presented as the mean ± SD. Levels of statistical significance for all data were determined by one-way ANOVA and Tukey test (* Indicates significant difference between the two groups; *p < 0.05; **p <0.01; ***p <0.001; ****p < 0.0001).

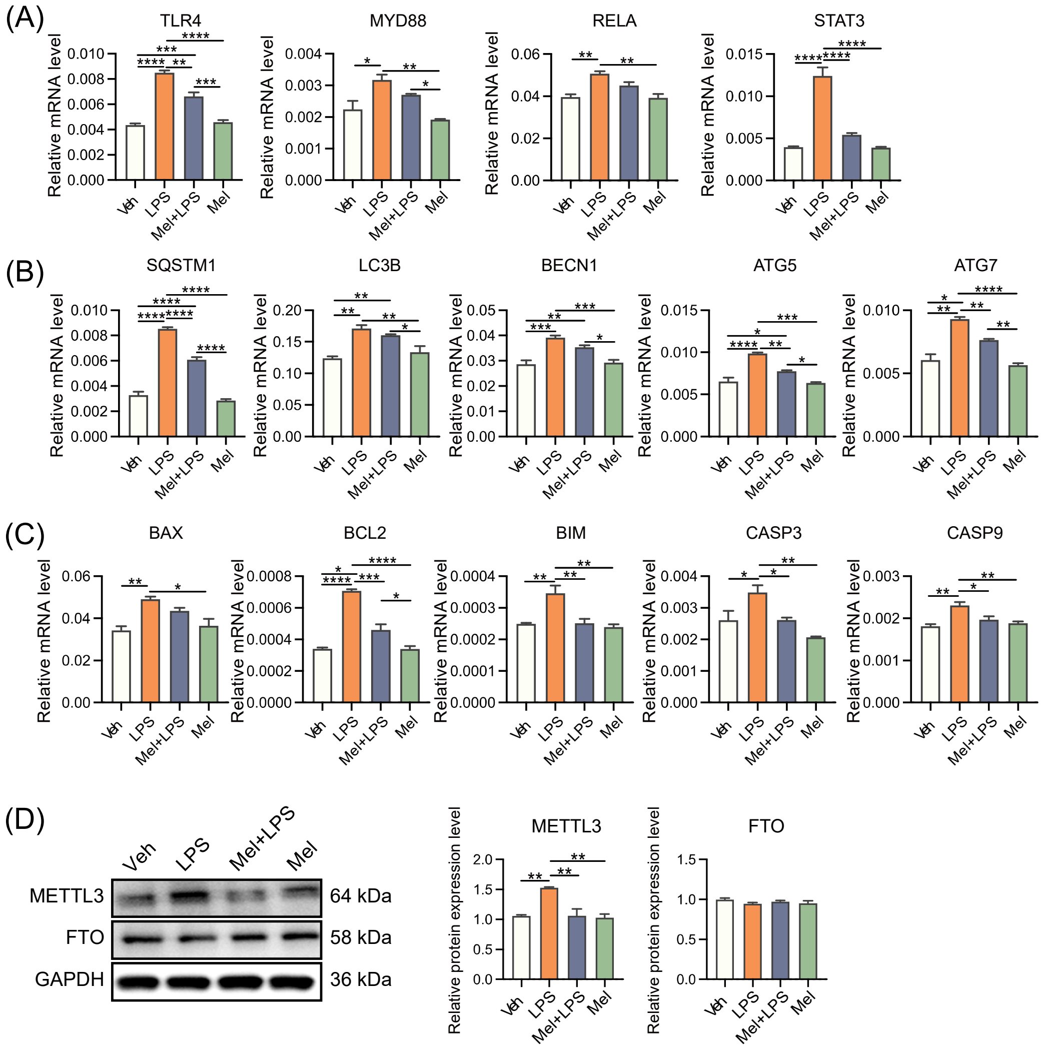

Figure S4 Transcript and protein levels detection in HESCs treated with melatonin and LPS.

(A) The mRNA levels of the inflammation-related genes in human endometrial stromal cells. n=3 independent biological replicates.

(B) The mRNA levels of the autophagy-related genes in human endometrial stromal cells. n=3 independent biological replicates.

(C) The mRNA levels of the apoptosis-related genes in human endometrial stromal cells. n=3 independent biological replicates.

(D) Western blot bands of METTL3 and FTO in human endometrial stromal cells treated with LPS and melatonin. n=3 independent biological replicates.

Veh: Vehicle treatment group; LPS: LPS treatment group; Mel+LPS: Melatonin and LPS co-treatment group; Mel: Melatonin treatment group; The data are presented as the mean ± SD. Levels of statistical significance for all data were determined by one-way ANOVA and Tukey test (* Indicates significant difference between the two groups; *p < 0.05; **p <0.01; ***p <0.001; ****p < 0.0001).

Table S1 Primers for mouse used in this study.

Transcript	Accession number		Primer sequences (5' to 3')
Gapdh	NM_001289726.1	Sense:	AGTCAAGGCCGAGAATGGGAAG
		Antisense:	AAGCAGTTGGTGGTGCAGGATG
Mettl3	NM_019721.2	Sense:	AAGGAGCCGGCTAAGAAGTC
		Antisense:	TCACTGGCTTTCATGCACTC
Mettl14	NM_201638.2	Sense:	CTGAGAGTGCGGATAGCATTG
		Antisense:	GAGCAGATGTATCATAGGAAGCC
Mettl16	NM_026197.3	Sense:	GACAAACCACCTGACTTCGCA
		Antisense:	TCTGACTGCTTCGGGGTCTT
Fto	NM_011936.2	Sense:	GCAGAGCAGCCTACAACGTGAC
		Antisense:	CCAACATGCCAAGTATCAGGATCTC
Alkbh5	NM_172943.4	Sense:	ACAAGATTAGATGCACCGCG
		Antisense:	TGTCCATTTCCAGGATCCGG
Ythdf1	NM_173761.3	Sense:	CATTATGAGAAGCGCCAGGA
		Antisense:	AGATGCAACAATCAACCCCG
Ythdf2	NM_145393.4	Sense:	ACCAACTCTAGGGACACTCA
		Antisense:	GGATAAGGAGATGCAACCGT
Tlr4	NM_021297.3	Sense:	GCCTTTCAGGGAATTAAGCTCC
		Antisense:	GATCAACCGATGGACGTGTAAA
Myd88	NM_010851.3	Sense:	ACCTGTGTCTGGTCCATTGCCA
		Antisense:	GCTGAGTGCAAACTTGGTCTGG
Rela	NM_009045.5	Sense:	ACTGCCGGGATGGCTACTAT
		Antisense:	TCTGGATTCGCTGGCTAATGG
Stat3	NM_213660.3	Sense:	GGGCCATCCTAAGCACAAAG
		Antisense:	GGTCTTGCCACTGATGTCCTT
Sqstm1	NM_011018.3	Sense:	GAACTCGCTATAAGTGCAGTGT
		Antisense:	AGAGAAGCTATCAGAGAGGTGG
Becn1	NM_019584.4	Sense:	ATGGAGGGGTCTAAGGCGTC
		Antisense:	TCCTCTCCTGAGTTAGCCTCT
Atg5	NM_053069.6	Sense:	TGTGCTTCGAGATGTGTGGTT
		Antisense:	ACCAACGTCAAATAGCTGACTC
Atg7	NM_028835.5	Sense:	TGACCTTCGCGGACCTAAAGA
		Antisense:	CCCGGATTAGAGGGATGCTC
Bcl2	NM_009741.5	Sense:	GCTACCGTCGTGACTTCGC
		Antisense:	CCCCACCGAACTCAAAGAAGG
Bax	NM_007527.3	Sense:	AGGATGCGTCCACCAAGAAGCT
		Antisense:	TCCGTGTCCACGTCAGCAATCA
Casp3	NM_009810.3	Sense:	CTGACTGGAAAGCCGAAACTC
		Antisense:	CGACCCGTCCTTTGAATTTCT
Casp9	NM_015733.5	Sense:	GCTGTGTCAAGTTTGCCTACCC
		Antisense:	CCAGAATGCCATCCAAGGTCTC

Table S2 Primers for human used in this study.

Transcript	Accession number		Primer sequences (5' to 3')
GAPDH	NM_002046.7	Sense:	GAAGGTGAAGGTCGGAGT
		Antisense:	GATGGCAACAATATCCACTT
METTL3	NM_019852.5	Sense:	TTGTCTCCAACCTTCCGTAGT
		Antisense:	CCAGATCAGAGAGGTGGTGTAG
METTL14	NM_020961.4	Sense:	GAACACAGAGCTTAAATCCCCA
		Antisense:	TGTCAGCTAAACCTACATCCCTG
FTO	NM_001080432.3	Sense:	CCAGAACCTGAGGAGAGAATGG
		Antisense:	CGATGTCTGTGAGGTCAAACGG
ALKBH5	NM_017758.4	Sense:	CCAGCTATGCTTCAGATCGCCT
		Antisense:	GGTTCTCTTCCTTGTCCATCTCC
YTHDF1	NM_017798.4	Sense:	ATACCTCACCACCTACGGACA
		Antisense:	GTGCTGATAGATGTTGTTCCCC
YTHDF2	NM_016258.3	Sense:	GTTGGTAGCGGGTCCATTACT
		Antisense:	GGTCTTCAGTTTAGGTTGCTGT
TLR4	NM_003266.4	Sense:	AGACCTGTCCCTGAACCCTAT
		Antisense:	CGATGGACTTCTAAACCAGCCA
MYD88	NM_002468.5	Sense:	GGCTGCTCTCAACATGCGA
		Antisense:	CTGTGTCCGCACGTTCAAGA
RELA	NM_021975.4	Sense:	ATGTGGAGATCATTGAGCAGC
		Antisense:	CCTGGTCCTGTGTAGCCATT
STAT3	NM_139276.3	Sense:	CAGCAGCTTGACACACGGTA
		Antisense:	AAACACCAAAGTGGCATGTGA
SQSTM1	NM_003900.5	Sense:	AAGCCGGGTGGGAATGTTG
		Antisense:	CCTGAACAGTTATCCGACTCCAT
LC3B	NM_022818.5	Sense:	GATGTCCGACTTATTCGAGAGC
		Antisense:	TTGAGCTGTAAGCGCCTTCTA
BECN1	NM_003766.5	Sense:	CCATGCAGGTGAGCTTCGT
		Antisense:	GAATCTGCGAGAGACACCATC
ATG5	NM_004849.4	Sense:	AAAGATGTGCTTCGAGATGTGT
		Antisense:	CACTTTGTCAGTTACCAACGTCA
ATG7	NM_006395.3	Sense:	ATGATCCCTGTAACTTAGCCCA
		Antisense:	CACGGAAGCAAACAACTTCAAC
BCL2	NM_138764.5	Sense:	ATCGCCCTGTGGATGACTGAGT
		Antisense:	GCCAGGAGAAATCAAACAGAGGC
BAX	NM_138764.5	Sense:	TCAGGATGCGTCCACCAAGAAG
		Antisense:	TGTGTCCACGGCGGCAATCATC
BIM	NM_138622.4	Sense:	TAAGTTCTGAGTGTGACCGAGA

		Antisense:	GCTCTGTCTGTAGGGAGGTAGG
CASP3	NM_032991.3	Sense:	AGAGGGGATCGTTGTAGAAGTC
		Antisense:	ACAGTCCAGTTCTGTACCACG
CASP9	NM_001229.5	Sense:	CTTCGTTTCTGCGAACTAACAGG
		Antisense:	GCACCACTGGGGTAAGGTTT

Table S3 Antibodies used in this study.

Antibody	From
Rabbit anti-METTL3 polyclonal antibody	Proteintech
Rabbit anti-FTO polyclonal antibody	Proteintech
Rabbit anti-MTNR1B polyclonal antibody	Abcam
Mouse anti-GAPDH monoclonal antibody	Proteintech
Rabbit anti-RELA monoclonal antibody	Abclonal
Rabbit anti-phospho-RELA polyclonal antibody	Abclonal
Rabbit anti-ERK1/2 polyclonal antibody	Abmart
Rabbit anti-PARP1 polyclonal antibody	Cell Signaling Technology
Rabbit anti-BAX polyclonal antibody	Proteintech
Rabbit anti-Caspase1 polyclonal antibody	Proteintech
Rabbit anti-Cleaved-Caspase3 polyclonal antibody	Cell Signaling Technology
Rabbit anti-LC3B polyclonal antibody	Novus
Rabbit anti-ATG5 polyclonal antibody	Proteintech
Rabbit anti-ATG7 polyclonal antibody	Proteintech
